# Optimum phosphate ion removal from aqueous solutions using roller kiln industrial solid waste

**DOI:** 10.1038/s41598-024-53962-9

**Published:** 2024-02-18

**Authors:** Dalia A. Ali, Walaa A. Abdelwahab, Mai H. Roushdy

**Affiliations:** Department of Chemical Engineering, The British University in Egypt, El-Sherouk City, 11837 Egypt

**Keywords:** Pollution remediation, Chemical engineering

## Abstract

Water scarcity is the most imperative predicament that concerns the population. In this research, a roller kiln (RK) industrial solid waste was used in the adsorption of phosphate ions from aqueous solutions thus converting a waste to wealth through aiding in serving as a water treatment application. The RK waste was produced from an Egyptian factory with a flow rate of million tons/day. Surface characterization for this solid waste was performed including transmission electron microscopy (TEM), X-ray diffraction (XRD), X-ray fluorescence (XRF), Fourier transform infra-red (FTIR), zeta potential (ZP), and particle size distribution (PSD). Based on the kinetics and isotherm studies, the pseudo first order (PFO) kinetic model and Freundlich isotherm model were the best-fitted models with the experimental data as well as the Dubinin–Radushkevich isotherm model indicated that the adsorption type was physical. The attained experimental results were then optimized to attain the experimental conditions at which the optimum adsorption percentage was achieved using response surface methodology (RSM). The optimum percentage removal of phosphate ions 99.5 (%) was achieved at the following experimental conditions; pH 8, temperature = 25 °C, contact time = 9 min, initial phosphate ion concentration = 10 mg/L and adsorbent dose 0.5 = g/L.

## Introduction

Industrial and domestic wastewater have many different compounds and contaminations. Therefore, wastewater treatment is necessary to ensure high quality of water and to eliminate the contaminants at a permissible limit according to the environmental regulations^1^. One of the substances found in nature like food, water, and human bodies is phosphorus which is an important nutrient for humans, plants, and animals^2^. The presence of high concentrations of phosphorous in water causes severe harmful effects on human health and the environment^3,4^. Phosphorous is a very reactive and non-metallic element that exists in three forms which are: white or yellow, black, and red^5^. Pure phosphorus is toxic^5^. There are many natural sources of phosphorus which are: water, animals, and plants^5^. While, the unnatural source of phosphorus is fertilizer^3^. Phosphorous exists in the form of phosphate^6^. Table 1 represents the numerical range of phosphorus content in the different sources and their allowable limit for safe disposal according to the Egyptian environmental regulations^7^ as water samples from the Nile river have phosphate ion concentration ranges from 0.025 to 0.311 mg/L which are below the permissible limit of pollution (1 mg/L)^8^. In El Khashab and El Hager canals, all water samples have phosphate ions concentration ranges from 0.246 to 1.298 mg/L. The collected samples 4 and 6 are polluted as they have concentrations more than the permissible limit of pollution (1.185 mg/L and 1.298 mg/L, respectively). The relatively high concentration of phosphate ion in the canals is attributed to the disposal of wastewater in them at these sites^8^. In El Saff wastewater canal, the phosphate ions concentrations samples range from 0.015 mg/L to 1.688 mg/L. The relatively high concentration of phosphate is detected at samples 9 and 10 as they have phosphate concentrations 0.988 and 1.688 mg/L, respectively, due to the outlet of drainage irrigation water into this canal, where the wastewater effluents represent important sources of phosphate^8^. In the groundwater samples, phosphate ion concentration ranges from 0.008 to 5.287 mg/L, the relatively high phosphate concentrations in groundwater samples are detected at samples 18 and 21 as their concentrations are 5.287 and 2.208 mg/L, respectively, more than the permissible limit of pollution referred to the seepage from El Saff wastewater canal at these localities and return flow after irrigation^8^.

**Table 1:** Concentrations of phosphate ion in the water resources samples within the study area (mg/L)^8^.

Surface water	Sample no.	PO_4_^3−^ (Permissible level = 1 mg/L)
Nile river	1	0.044
	2	0.311
	3	0.025
El-Khashab and El-Hager canals	4	1.185
	5	0.356
	6	1.298
	7	0.246
El-Saff wastewater canal	8	0.442
	9	0.988
	10	1.688
	11	0.015
	12	0.299
	13	0.134
Groundwater	14	0.008
	15	0.015
	16	0.442
	17	0.09
	18	5.287
	19	0.016
	20	0.219
	21	2.208
	22	0.207
	23	0.071
	24	0.08
	25	0.140
	26	0.093
	27	0.120
	28	0.091
	29	0.091
	30	0.218
	31	0.292
	32	0.11
	33	0.2
	34	0.25
	35	0.403

There are different methods used for the removal of phosphorous from wastewater including biological, chemical precipitation, electrocoagulation, and adsorption methods^9^. The biological method removes phosphorous from wastewater in high percentages reaching 97% but, with high energy consumption and production of huge amounts of sludge waste which need furthermore treatment^5,10^. Chemical precipitation is another common method used to remove phosphorous from wastewater by using coagulants like calcium, iron, and aluminum, although this method removes phosphorous successfully from wastewater in high percentages^11^, it has disadvantages represented in large consumption of chemicals and production of other undesired solid wastes which need furthermore treatment^5,10^. Electrocoagulation is a chemical method which involves the use of electric current with electrodes made of iron and aluminum which cause the colloidal contaminants to coagulate like phosphate ions and produce gas bubbles^5,10^. The phosphate exists in this wastewater treatment method has a negative charge which leads to the formation of electrical double layers^9^. There is an electrostatic attraction between the pollutants that have negative charge and the hydrolysis product that has positive charge resulting in a destabilization of the electrical double layer and neutralization^9,11^. After that flocculation process is accomplished, a sedimentation process is done to remove the pollutants (phosphate ions) by settling them down^10^. Adsorption method is commonly used due to the availability of different types of adsorbents, cheapness, and high adsorption capacity even at low phosphate concentrations^12^. Many natural and synthetic adsorbents can be used for phosphate ions removal from wastewater such as coal fly ash, polymer hydrogels and industrial solid wastes like Fume Dust from Electric Arc Furnace (FD-EAF)^13^. In the crystallization process calcium reacts with hydroxide ions that exist in the aqueous alkaline solution to remove phosphate ions in the form of hydroxyapatite^10,14^. Due to the hydrophilic and three-dimensional polymeric network of polymer hydrogel, it is observed that polymer hydrogel is effective to be used as an adsorbent for phosphate ion removal from wastewater^9,14–16^. Because, nanomaterials/nanoparticles, snail shell dust and, carbon-based materials (activated carbon and biochar) have exceptionally high surface areas and phosphate adsorption properties, they are widely used for phosphate removal from wastewater^11,17–23^. In this research an industrial solid waste called roller kiln (RK) is used for phosphate ion removal from aqueous solutions. It is produced from an Egyptian ceramic factory in Egypt with a flowrate of million tons/day.

## Chemicals and methods

### Chemicals

Roller kiln (RK) solid waste was supplied from ceramic factory, Egypt. Analytical grade reagents were used in this study, including, Sodium Phosphate Monobasic (NaH_2_PO_4_), Ascorbic Acid, Ammonium Molybdate ((NH_4_)_6_Mo_7_O_24_) and concentrated Sulfuric acid (H_2_SO_4_ 96%). These reagents were purchased from Morgan Chemical Ltd., Egypt. All solutions were prepared using double-distilled water.

### Equipment used in characterization of the Roller Kiln (RK) solid waste

Transmission Electron Microscope (TEM) (JEM-1400Flash, JEOL Solutions for Innovation Company, USA) was used to illustrate clearly the morphology of the RK solid waste into scaling approaching 1 nm^24^. An Empyrean—Malvern Analytical—Netherland diffractometer with Ni-filtered Cu k radiation (40 kV, 30 mA, = 1.5406°A) was used to perform X-ray diffraction (XRD) on RK solid waste to illustrate its purity and degree of crystallinity^25^. The samples were analyzed using an XRD over a range of 5.009°–99.987°. Fourier-transform infrared (FTIR) analysis (Vertex 70 RAM II, Germany) was used to record the surface functional groups of RK solid waste^25^. The surface charge of the RK industrial solid waste was detected using dynamic light scattering instrument (ZetaSizer Nano Series (HT), Nano ZS, Malvern Instruments, UK)^26,27^. X-ray fluorescence (XRF) analysis was conducted to examine each oxide quantity contained in the RK solid waste^28,29^ utilizing American Society for Testing and Materials (ASTM) recommendations (C114-18). The particle size distribution (PSD) was determined by employing a set of standard screens with standard openings utilizing the standard ASTM D 422/2007 for the method and ASTM E 11/2009 for the sieves.

### Batch experiments

The efficiency of the synthesized RK solid waste in the removal of the phosphate ion was determined using batch experiments with three influencing factors: Based on the Egyptian Environmental Law number 4 of 1994, the initial phosphate ion concentration was chosen in the range of 1–10 mg/L^7^, adsorbent dose (0.5 g/L to 6.5 g/L), and contact period (6 min to 90 min). Experiments were carried out at pH 8 and temperature = 25 °C in glass conical flasks that were forcefully shaken at 200 rpm. Using a centrifuge (D-37520 Osterode, Kendro Laboratory Products Company), samples were separated after shaking. A standard colorimetric method^30^ and a UV/VIS spectrophotometer (UV-5100, Shanghai Metash Instruments Company) were used to determine the concentration of phosphate ion in the filtered solution. The phosphate ion removal efficiency was obtained using Eq. (1)^31^:

1 $${\text{RE}}\%=[({\text{C}}_{\rm o} - {\text{C}})/{\text{C}}_{\rm o}]*100$$

where C_o_ and C are initial and final phosphate ion concentrations in mg/L, respectively.

### Statistical tests

The Sum of Squared Errors (SSE) was calculated for each isotherm model to determine which is the best-fit by non-linear regression based on the following Eq. (2)^32,33^:

2 $${\text{SSE}}= \sum_{{\text{i}}=1}^{{\text{n}}}{({{\text{q}}}_{{\text{e}},{\text{exp}}} - {{\text{q}}}_{{\text{e}},{\text{calc}}})}^{2}$$

where q_e,exp_ (mg/g) is the experimental adsorption capacity at equilibrium and q_e,calc_ (mg/g) is the calculated adsorption capacity at equilibrium.

## Results and discussion

### Characterization of the Roller Kiln (RK) solid waste

#### Transmission electron microscope (TEM) investigation

**Figure 1:**
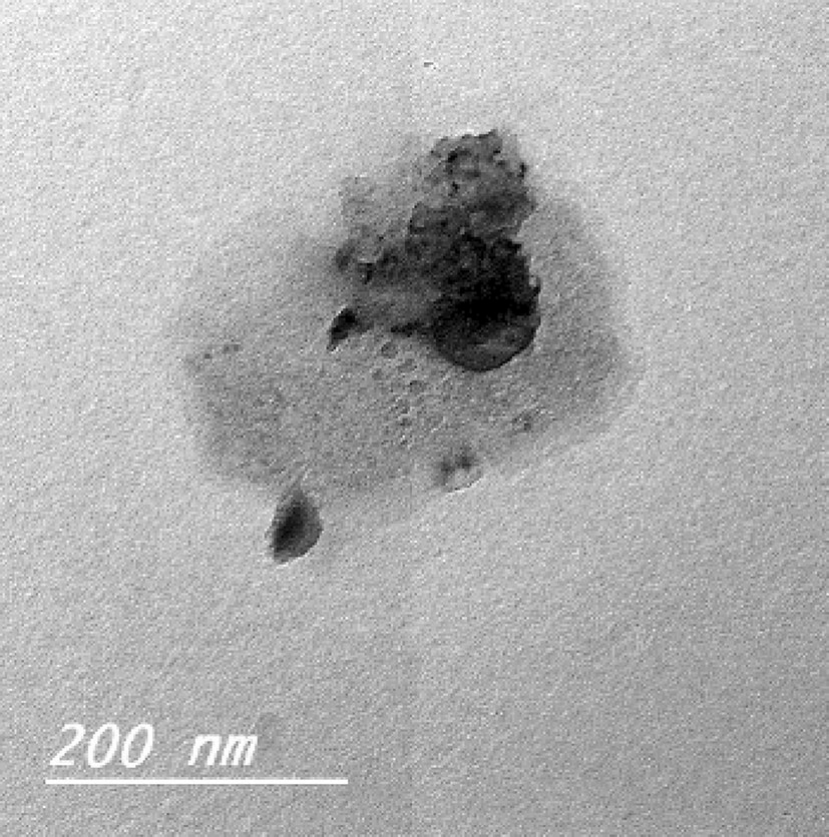
Nano-sized particles of roller kiln waste powder.

TEM was used to obtain clear micrographs of single particles of the roller kiln solid waste powder. Figure 1 suggested the presence of core – shell structures within the powder. The probable configuration consisted of a multi- core within an alumina shell.

**Figure 2:**
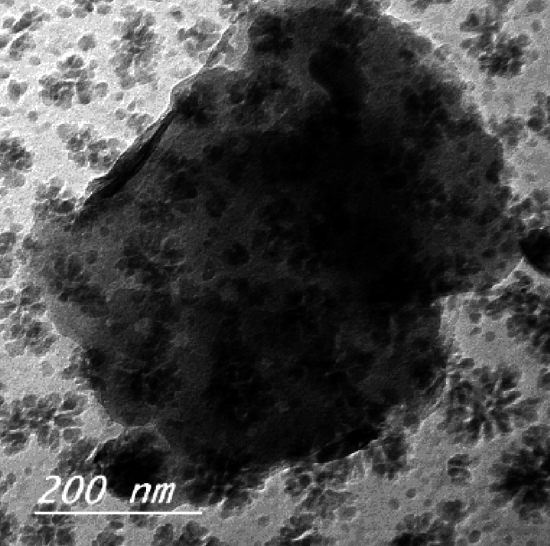
Rosette structure of roller kiln waste powder.

**Figure 3:**
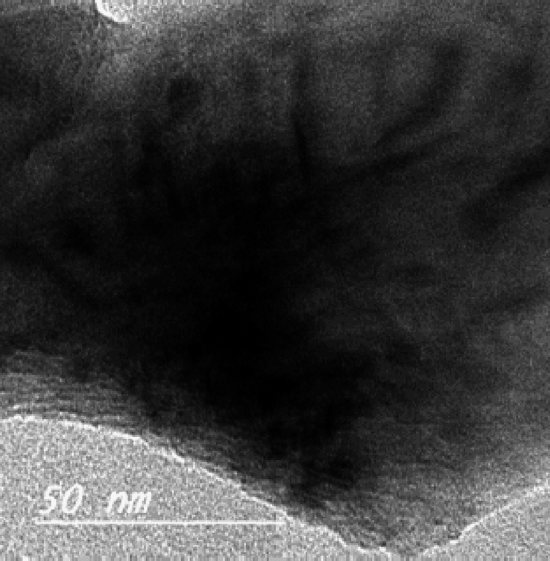
Development of dendrites within roller kiln waste powder particles.

When coarser particles were observed in Fig. 2, rosette-like segregations could be seen. It was made up of a variety of branches. This was due to crystals with the same form growing from random solid inclusions in the interior. These inclusions were typically caused by little amounts of glaze spilling on the roller surface as the tiles travel through the kiln. These crystals generated rosette-like, nearly spherical crystals as a result of grain development. The consistent temperature to which a roller was exposed during kiln operation favored this arrangement^34,35^. When a roller was removed from the kiln to be ground to remove surface inclusions, the grain growth mechanism stopped, allowing some dendritic structure to form, as shown in some micrographs. The presence of anorthite phase caused the dendritic structure to develop (Fig. 3).

#### X-ray fluorescence (XRF)

The chemical analysis of the used RK solid waste was represented in Table 2. Alumina *(*Al_2_O_3_) was the main component in roller kiln waste 83.75*%* then silica (SiO_2_) with 10.69% and the remainings were negligible amounts of other oxides. Also, the loss of ignition (LOI) value was almost zero.

**Table 2:** Chemical analysis of roller kiln solid waste.

Main constituents	Roller kiln waste
SiO_2_	10.69
Al_2_O_3_	83.75
Fe_2_O_3_	1.61
TiO_2_	0.21
ZrO_2_	2.35
MnO	< 0.01
SO_3_	0.07
MgO	0.22
CaO	0.25
Na_2_O	0.02
K_2_O	0.03
Cl	0.06
P_2_O_5_	0.03
LOI	0.42

#### X-ray diffraction (XRD)

**Figure 4:**
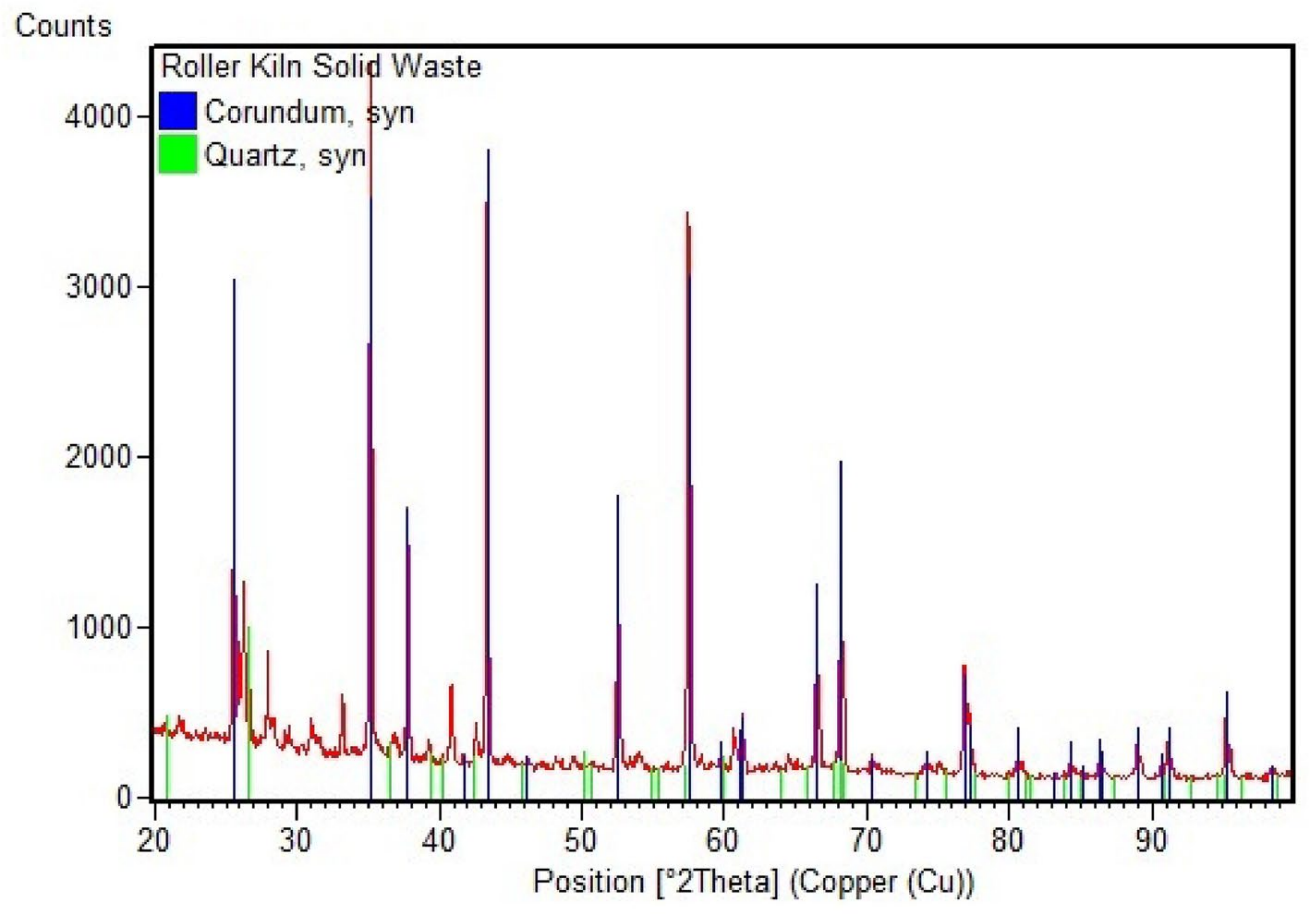
XRD pattern of roller kiln solid waste before adsorption.

Figure 4 represented that the main phase in the RK solid waste was Corundum. Corundum is the crystalline form of Aluminium Oxide (Al_2_O_3_). It was worth mentioning that there were other phases which were Mullite (Al_1.272_Si_0.728_O_4_._864_), Anorthite (CaAl_2_Si_2_O_8_) and Gehlenite (Ca_2_Al((Al Si) O_7_).

**Figure 5:**
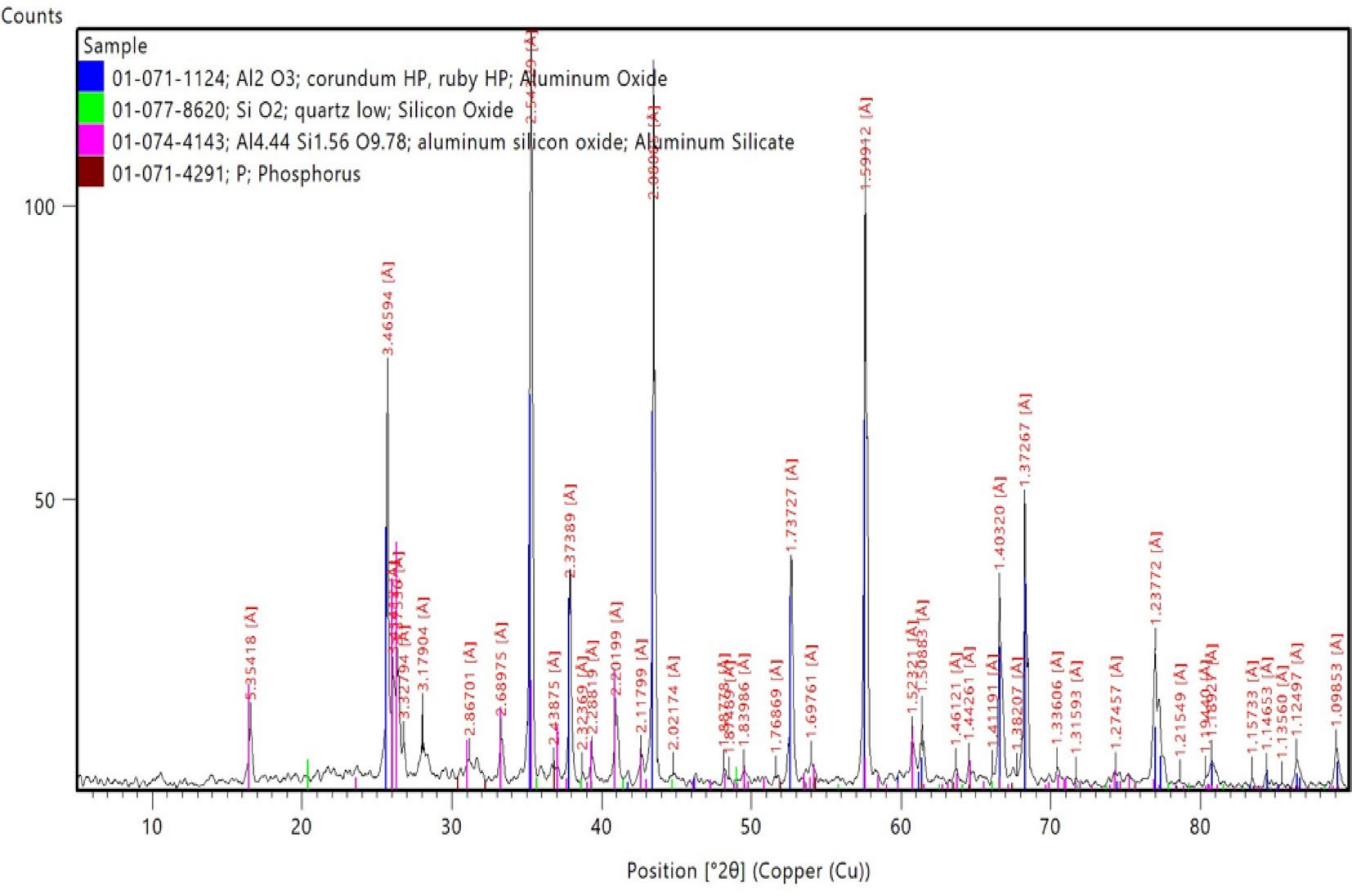
XRD pattern of roller kiln solid waste after adsorption.

Figure 5 represented the XRD analysis of the RK solid waste after the adsorption of phosphate ions from an aqueous solution. New peaks appeared corresponding to the phosphorous that ensured the successful adsorption of the phosphate ion using the RK industrial solid waste.

#### Particle size distribution (PSD)

**Figure 6:**
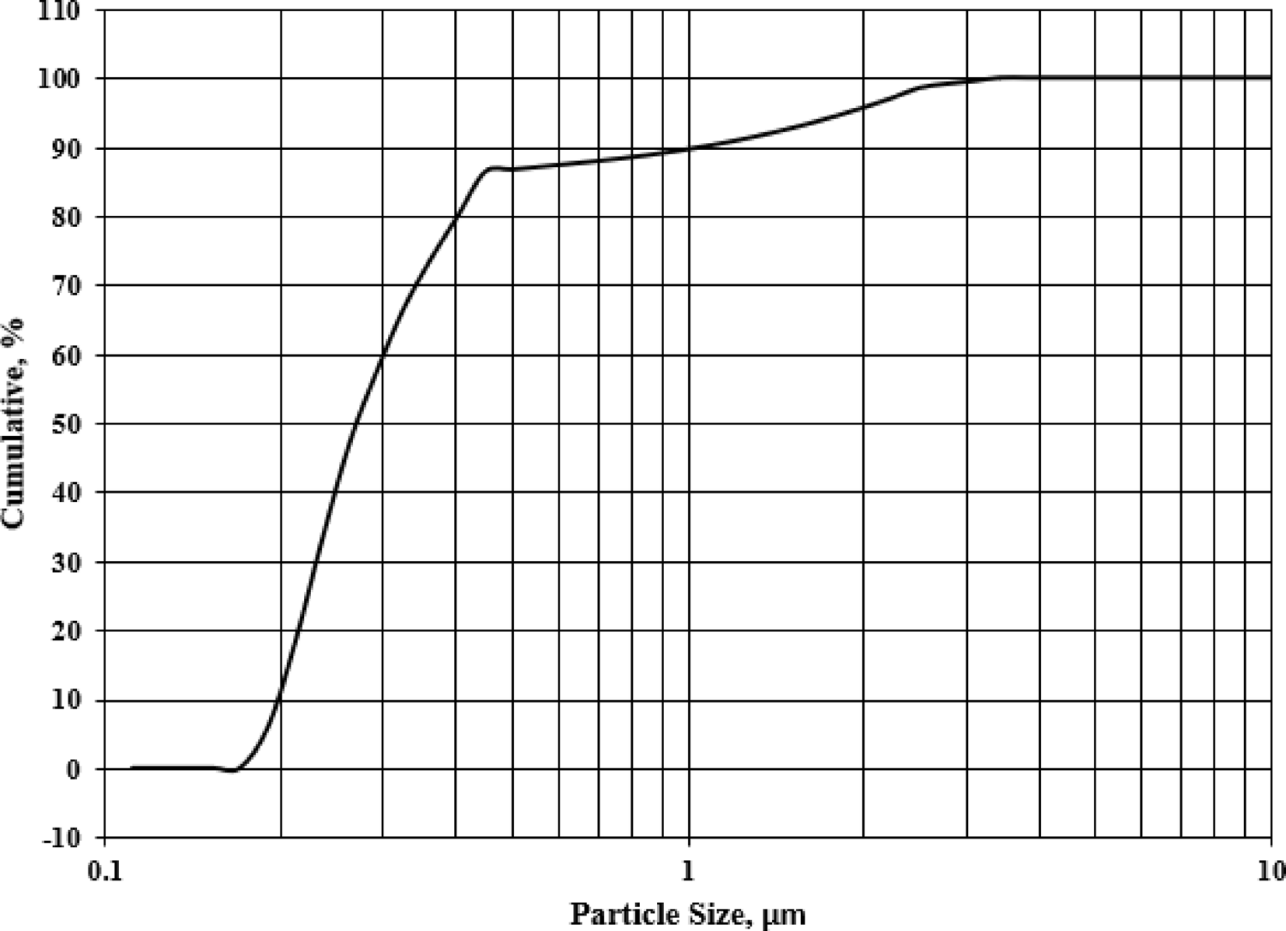
Nano-sized particles of roller kiln solid waste powder.

Figure 6 represented the cumulative screen analysis curve of RK solid waste. The fraction retained on each diameter screen was represented on the vertical axis. It was observed that the grind waste was fine. Different percentages of RK solid waste were used at different diameters of the cumulative screen. Mean particle size of the RK waste powder = 0.287 μm.

#### Fourier transform infrared (FTIR)

**Figure 7:**
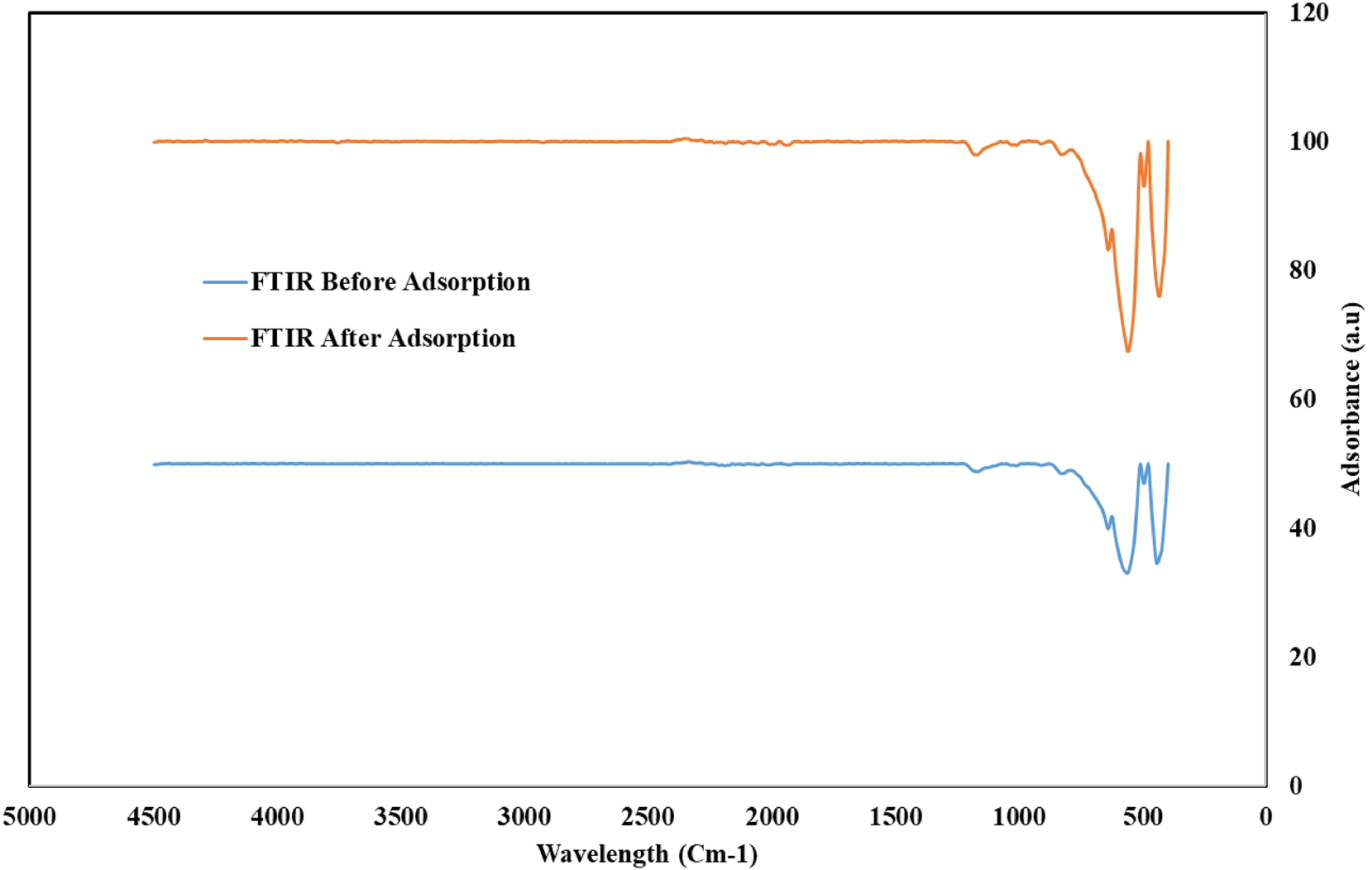
FTIR of roller kiln solid waste before and after the adsorption of phosphate ions from an aqueous solution.

Figure 7 represented the FTIR bands of the RK solid waste before and after adsorption of phosphate ion from aqueous solutions. Before adsorption; the sharp peaks at 442.15 cm^−1^, 495.66 cm^−1^, 563.21 cm^−1^ and 639.85 cm^−1^ were attributed to Al_2_O_3_^36^. The small broad peak at 1171.09 cm^−1^ corresponded to the Si–O^36^. It was observed that the intensity of Al_2_O_3_ and Si–O peaks had shifted slightly and increased after the adsorption process confirming successful phosphate ion adsorption onto the surface of the RK industrial solid waste.

#### Zeta potential (ZP)

**Figure 8:**
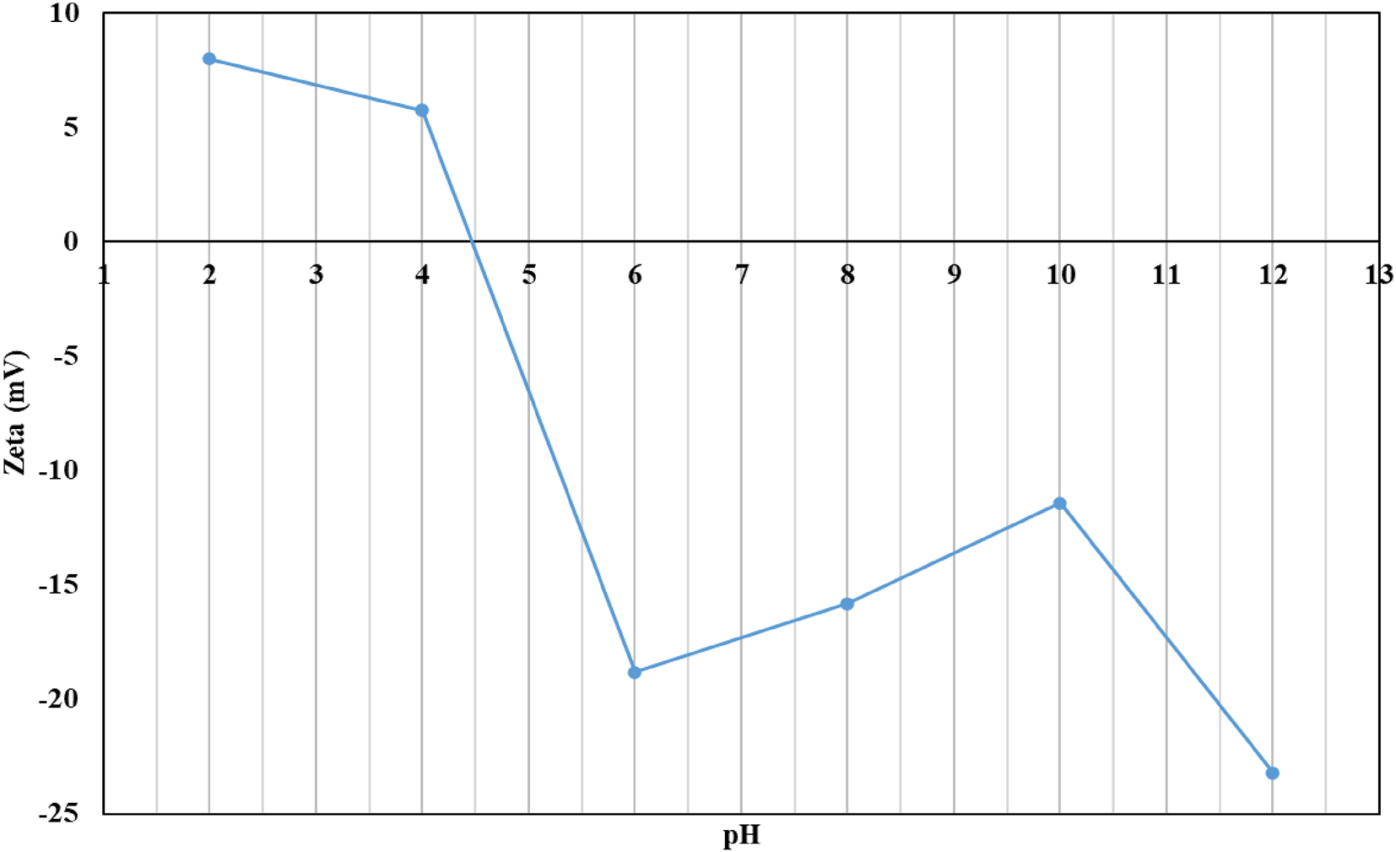
Zeta potential at different pH.

Figure 8 depicted the RK solid waste's point of zero charge (pH_pzc_) to be around 4.5. When the pH was below the pH_pzc_ (4.5), the surface of the RK solid waste was positively charged, allowing it to adsorb anionic contaminants efficiently in this pH range. Whenever the pH was above pH_pzc_ (4.5), the surface of the RK waste was negatively charged and could readily adsorb cationic contaminants. This ensured that the RK solid waste was capable of adsorbing in both acidic and basic environments.

### Analysis of the phosphate ion adsorption from aqueous solutions

**Figure 9:**
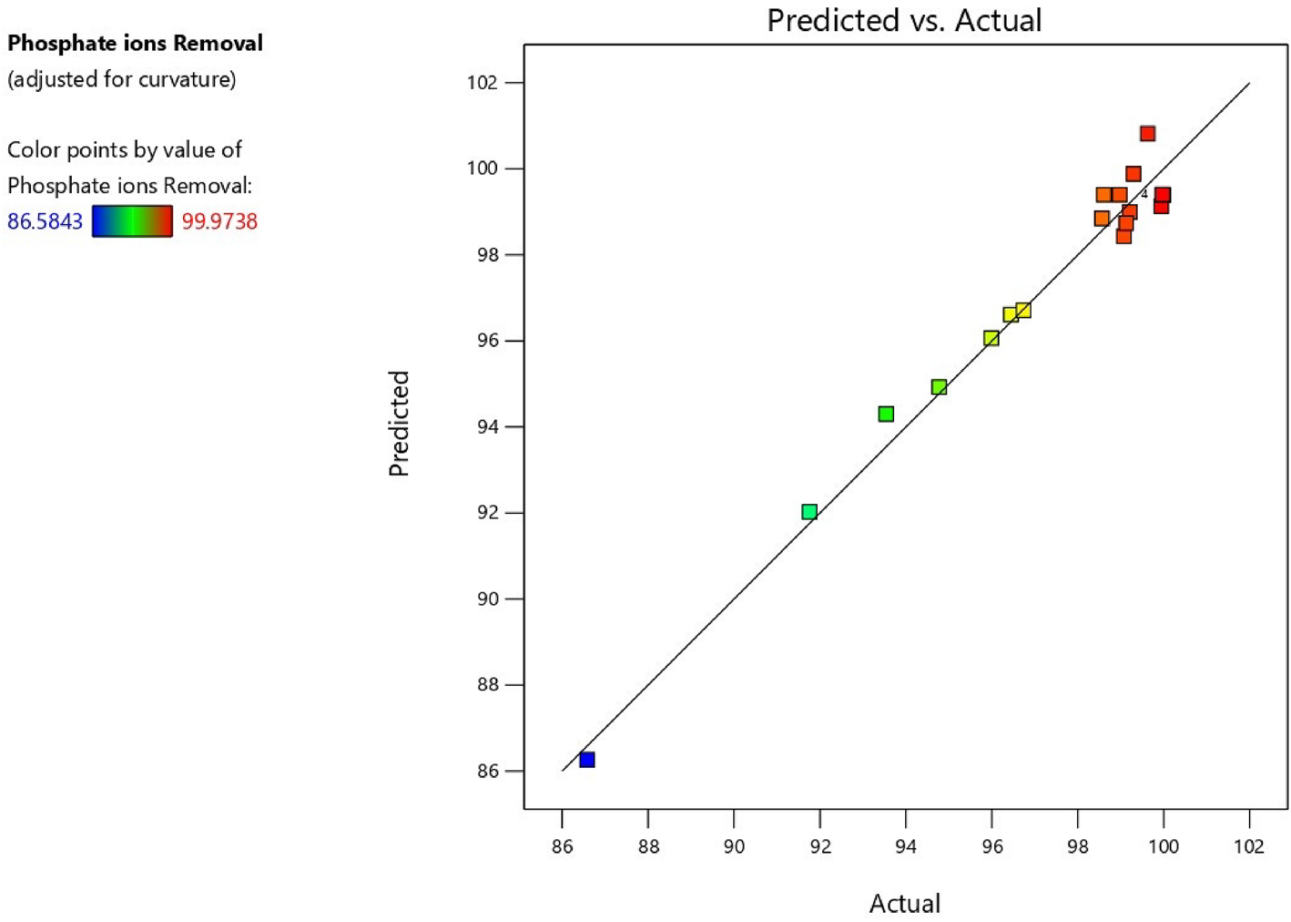
Relation between the actual and predicted removal efficiency.

The adsorption experiments were performed to determine the phosphate ions removal percents at the following conditions of initial phosphate ion concentration in a range of 1 – 10 mg/L, contact time or adsorption time in a range of 6 – 90 min, adsorbent dose in a range of 0.5 – 6.5 g/L, pH 8 and temperature = 25 °C. Design Expert V13 generated models representing the relation between process parameters and process response which was the phosphate ions removal. Analysis of Variance (ANOVA) method was adopted at a confidence level of 95% in order to conclude whether the resulted models were significant and suitable or not through determining P and F values. The optimum significant model for phosphate ion removal was the quadratic model. There were several terms that weren’t significant enough in the model as their P values were higher than 0.1, and thus, the models were simplified to the reduced ones. The module was demonstrated in Eq. (3) and the results of the (ANOVA) analysis were summarized in Table 3. Furthermore, the calculated and experimental results for phosphate ion removal exhibited a reasonable agreement as confirmed by Fig. 9, and the values of adjusted and predicted R squared in Table 3. This agreement confirmed the adequacy of the models.

3 $${\text{X}}=92.57+1.74 {\mathrm{A}}-0.031 {\mathrm{B}} + 0.78 {\mathrm{C}} + 0.01 {\mathrm{AB}} - 0.21 {\mathrm{AC}}+0.01 {\mathrm{BC}}-{0.11 {\mathrm{A}}}^{2}-{0.00088 {\mathrm{B}}}^{2}$$

where (X) denotes phosphate ion removal efficiency, phosphate ion concentration (A), contact time or adsorption time (B) and quantity of adsorbent (C).

**Table 3:** Results of ANOVA analysis for phosphate ion removal response.

Source	Sum of Squares	Mean Square	p-value	
Model	223.71	27.96	< 0.0001	High Significant
A-Phosphate ions concentration	31.66	31.66	< 0.0001	High Significant
B-Contact time	0.5297	0.5297	0.03481	Significant
C-Amount of adsorbent	7.95	7.95	0.0030	Significant
AB	39.84	39.84	< 0.0001	High Significant
AC	61.57	61.57	< 0.0001	High Significant
BC	19.32	19.32	0.0001	Significant
A^2^	41.17	41.17	< 0.0001	High Significant
B^2^	16.89	16.89	0.0002	High Significant
R^2^	0.9736			
Adjusted R^2^	0.9544			
Predicted R^2^	0.8888

#### Variation of phosphate ion removal percentage with process conditions

**Figure 10:**
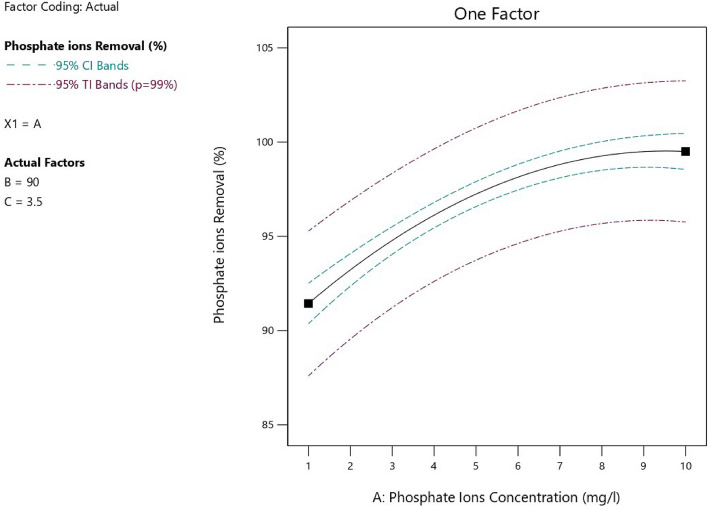
Relation between the phosphate ions concentration and the phosphate ion removal efficiency.

**Figure 11:**
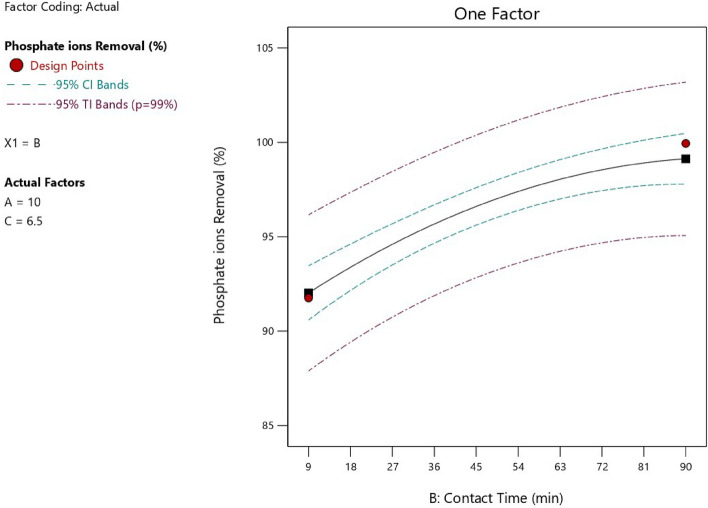
Relation between the contact time and the phosphate ion removal efficiency.

**Figure 12:**
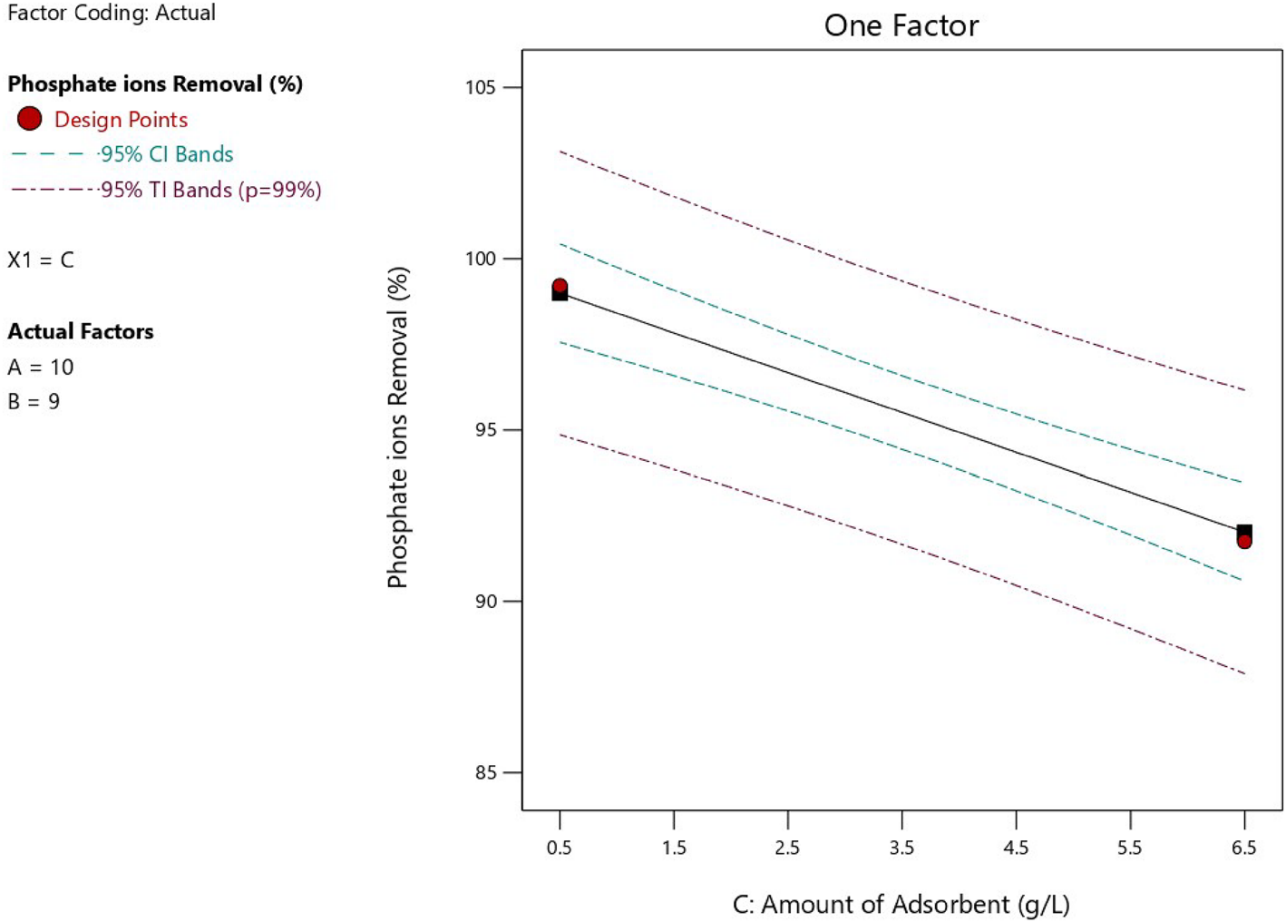
Relation between the adsorbent amount and the phosphate ion removal efficiency.

Figures 10, 11, and 12 represented the effect of each parameter of the process on the removal percentage of phosphate ion from aqueous solutions. All experimental parameters included initial phosphate ion concentration, contact time and the adsorbent dosage had high influence on the removal of phosphate ions from aqueous solutions. There was a direct relationship between the concentration of phosphate ions and the removal percentage of phosphate ions at experimental conditions of pH 8, temperature = 25 °C, adsorbent dose = 3.5 g/L and adsorption time = 90 min as represented in Fig. 10. Additionally, as represented in Table 4, it was observed that the adsorption efficiency increased from 94.5 to 99.97% with increase in the initial phosphate ion concentration from 1 mg/L to 5.5 mg/L at fixed experimental conditions of pH 8, temperature = 25 °C, adsorbent dose = 3.5 g/L and adsorption time = 50 min as the driving force increased, the resistance of mass transfer decreased, and consequently the process efficiency and the percentage of phosphate ions removal. While more increase in the initial phosphate ion concentration from 5.5 g/L to 13 g/L led to decrease in the phosphate ion removal percent from 99.97 to 90.98% due to deficiency in the available active sites with extra increase in the phosphate ion concentration.

**Table 4:** Effect of initial phosphate ion concentration on the phosphate ion removal percent.

Initial concentration of phosphate ion (mg/L)	1	2	3.5	5.5	10	13
Removal percent of phosphate ion (%)	94.5	96.73	98.42	99.97	98.94	90.98

As represented in Fig. 11, there was a direct relationship between the adsorption time and the removal percentage of phosphate ions at experimental conditions of pH 8, temperature = 25 °C, adsorbent dose = 6.5 g/L and initial phosphate ion concentration = 10 mg/L as there would be a sufficient time for separation. Moreover, Table 5 represented that the adsorption efficiency increased from 91.15 to 99.97% with increasing in adsorption time from 10 to 50 min at specified experimental conditions of pH 8, temperature = 25 °C, adsorbent dose = 3.5 g/L and initial phosphate ion concentration = 5.5 g/L. But, this occurred until reaching the equilibrium state at which the removal efficiency was almost constant at time range of 80 to 100 min.

**Table 5:** Effect of adsorption time on the phosphate ion removal percent.

Adsorption time (min)	10	20	30	40	50	80	100
Removal percent of phosphate ion (%)	91.15	93.3	95.5	98.2	99.97	99.93	99.9

As shown in Fig. 12, increasing adsorbent dose, increased the available specific surface area, which resulted in an increase in phosphate ion removal percentage when pH 8, temperature = 25 °C, adsorption time = 9 min, and initial phosphate ion concentration = 10 mg/L. For more illustration, Table 6 showed that the removal efficiency of phosphate ion increased from 90.4 to 99.97% with increasing in the adsorbent dose from 0.5 g/L to 3.5 g/L at specified experimental conditions of pH 8, temperature = 25 °C, adsorption time = 50 min, and initial phosphate ion concentration = 5.5 mg/L. But, this increase was to some extent as an extra increase in the adsorbent dose from 3.5 g/L to 12 g/L promoted the agglomeration of the particles which consequently reduced the specific surface area and eventually reduced the phosphate ion removal efficiency from 99.97 to 82.73%.

**Table 6:** Effect of adsorbent dose on the phosphate ion removal percent.

Adsorbent dose (g/L)	0.5	1.5	3.5	6.5	8.5	12
Removal percent of phosphate ion (%)	90.4	98.56	99.97	99.8	99.62	82.73

### Adsorption kinetics study

This kinetics study was performed using non-linear forms of Pseudo first Order (PFO) and Pesudo Second Order (PSO) models with non-linear regression using the Least Square method^37,38^. The kinetics of phosphate ion removal by RK solid waste were investigated at various contact durations ranging from 6 to 119 min and under the following fixed experimental conditions: pH 8, initial phosphate ion concentration = 5.5 mg/L, temperature = 25 °C, and adsorbent dosage = 3.5 g/L. The results in Table 7 represented that the PFO model was the best fitted model with the experimental data for phosphate ion adsorption from aqueous solutions utilizing RK solid waste because it had a lower SSE value (0.0003) than the PSO model (0.00057). Moreover, it was obviously observed that the calculated adsorption capacity at equilibrium (q_e-calculated_) was close to its experimental value (q_e-experimental_) which proved that PFO model was the best fitted model with rate constant (K_1_) of 0.781 min^−1^.

**Table 7:** The results of PFO and PSO kinetic models.

Kinetic models	Parameters	Values
PFO	q_e_ (experimental) (mg/g)	1.529
	q_e_(calculated) (mg/g)	1.553
	K_1_ (min^−1^)	0.781
	SSE	0.0003
PSO	q_e_ (experimental) (mg/g)	1.529
	q_e_(calculated) (mg/g)	2.529
	K_2_ (mg/g. min)	5.085
	SSE	0.00057

### Adsorption isotherm study

Adsorption isotherm study for this research was performed using the rules mentioned in literature^39,40^. Langmuir, Freundlich, and Dubinin–Radushkevich isotherm models were investigated at various phosphate ion concentrations ranged from 1 mg/L to 13.1 mg/L and under the following fixed experimental conditions: contact time = 90 min, pH 8, temperature = 25 °C, and adsorbent dosage = 3.5 g/L. Based on Table 8, Freundlich's isotherm model fit the experimental data better than Langmuir's isotherm model because the Freundlich's SSE value (0.0896) was lower than Langmuir's (0.7844). In addition, the separation factor (R_L_) was higher than 1 which indicated that this adsorption process was unfavourable to be represented by Langmuir isotherm model^41^. The constant K_L_ in Langmuir isotherm related to the energy of adsorption with value of 0.005 L/mg. While in the Freundlich isotherm, K_H_ constant indicated the adsorption capacity^42^ with a value of 33.495 (L/mg). Moreover, the index “1/n” indicated the intensity of adsorption or surface heterogeneity as when 0 < 1/n (= 0.608) < 1, adsorption was considered favorable^42^. The results of Dubinin–Radushkevich isotherm model indicated that the adsorption process involved physical adsorption between the surface of the RK solid waste and phosphate ions as evidenced by the value of the mean adsorption energy (E = 2.31 kJ/mol) < 8 kJ/mol^43^. Therefore, the adsorption system was considered to be physical with a multi-layer adsorption mechanism and a high adsorption capacity of phosphate ions (q_max_ = 2378.1 mg/g).

**Table 8:** The results of different isotherm models.

Isotherm models	Parameters	Values
Langmuir	SSE	0.7844
	q_max_ (mg/g)	2378.1
	K_L_ (L/mg)	0.005
	R_L_ (Separation factor)	1.358
Freundlich	SSE	0.0896
	1/n	0.608
	K_H_ (L/mg)	33.495
Dubinin–Radushkevich	β (mol^2^/kJ^2^)	0.086
	E (kJ/mol)	2.41
	q_max_ (mg/g)	13.43

### Process optimization

The phosphate removal process was optimized in order to get the best values for the independent variables (contact duration, phosphate ions concentration, and adsorbent quantity) that affect the dependent response variable (phosphate ion removal percentage). The Design Expert V13 software was used to create the numerical optimization step, which involved integrating the desirability of each independent variable into a single value and then searching for optimum values for the response goals. As a result, in order to determine the optimal conditions for the independent variables, a set of targets must be created on the aforementioned software to lead the optimization process. The independent variables' targets have been chosen based on environmental and economic concerns, as well as the aims presented in Table 6. Design Expert V13 software generated 50 suggested solutions with different desirability and then, the optimum solution with the highest desirability was selected. As represented in Table 9, the optimum phosphate ion removal percent (99.52%) was obtained at experimental conditions of pH 8, temperature = 25 °C, initial phosphate ion concentration = 10 mg/L, contact duration = 9 min and amount of adsorbent = 0.5 g/L.

**Table 9:** Optimization goals and results.

Reaction parameter/response	Goal	Resulted value
A: Phosphate ion concentration (mg/l)	Maximize	10
B: Contact time (min)	Minimize	9
C: Amount of adsorbent (g/L)	Minimize	0.5
Phosphate ion removal percentage	Maximize	99.52%

## Conclusion

Surface characterization was performed for the RK industrial solid waste including SEM, EDX, XRD, FTIR, PSD, zeta potential and XRF. The adsorption efficiency of phosphate ion from aqueous solutions was achieved successfully using the RK solid waste as represented in the XRD and FTIR analysis which were performed before and after adsorption as well as kinetics and isotherm studies were performed for this adsorption system to determine the adsorption type and mechanism. PFO kinetic model was the best fitted model with the experimental data. Freundlich isotherm was the best fitted model with the experimental data indicated that the system was a multi-layer adsorption with maximum adsorption capacity (q_max_) of 13.43 mg/g as well as Dubinin–Radushkevich model indicated that the type of this adsorption system was physical. Optimization for this adsorption process was performed using the Design Expert software (version 13) based on Response Surface Methodology (RSM) to determine the optimum phosphate ion removal efficiency from aqueous solutions. This optimum removal percentage (99.52%) was achieved at experimental conditions of pH 8, temperature = 25 °C, contact time = 9 min, initial phosphate ion concentration = 10 mg/L and adsorbent dose = 0.5 g/L.

## Data Availability

The data used to support this study's findings are available from the corresponding author upon request.

## References

[1] Kesari, K. K., Soni, R., Mohammad, Q. & Jamal, S. Wastewater treatment and reuse: A review of its applications and health implications. (2021).

[2] Ge X (2023). Toward a better understanding of phosphorus nonpoint source pollution from soil to water and the application of amendment materials: Research trends. Water.

[3] Bunce JT, Ndam E, Ofiteru ID, Moore A, Graham DW (2018). A review of phosphorus removal technologies and their applicability to small-scale domestic wastewater treatment systems. Front. Environ. Sci..

[4] Durán-Sánchez A, Álvarez-García J, del Río-Rama MC (2018). Sustainable water resources management: A bibliometric overview. Water.

[5] Yasipourtehrani S, Strezov V, Evans T (2019). Investigation of phosphate removal capability of blast furnace slag in wastewater treatment. Sci. Rep..

[6] De-Bashan LE, Bashan Y (2004). Recent advances in removing phosphorus from wastewater and its future use as fertilizer (1997–2003). Water Res..

[7] LAW NUMBER 4 of 1994*. *Environment* (1994). 10.1002/hep.27530.

[8] El-Aassar A (2017). Pollutants detection in water resources at El Saff area and their impact on humanhealth. Giza Governorate, Egypt..

[9] Arambarri J, Abbassi B, Zytner P (2019). Enhanced removal of phosphorus from wastewater using sequential electrocoagulation and chemical coagulation. Water. Air. Soil Pollut..

[10] Hoon, C. H. The removal methods of phosphorus/phosphate and nitrogen/nitrate from water and wastewater. 8–90 (2012).

[11] Ren J, Li N, Wei H, Li A, Yang H (2020). Efficient removal of phosphorus from turbid water using chemical sedimentation by FeCl_3_ in conjunction with a starch-based flocculant. Water Res..

[12] Usman MO, Aturagaba G, Ntale M, Nyakairu GW (2022). A review of adsorption techniques for removal of phosphates from wastewater. Water Sci. Technol..

[13] Ali DA, Al-Mansi NM, Sadek MA, Aboelnasr AW (2021). Simultaneous removal of nitrate and phosphate ions from aqueous solutions using fume dust from electric arc furnace industrial waste. Chem. Eng. Trans..

[14] Cheng R, Qiu L, Lu L, Hu M, Tao Z (2017). Research and application of crystallization technology in treating phosphorus wastewater..

[15] Mahmoud GA, Khalek MAA, Shoukry EM, Amin M, Abdulghany H (2019). Removal of phosphate ions from wastewater by treated hydrogel based on chitosan. Egypt. J. Chem..

[16] Oktor K, Yenihan N (2023). Optimization of removal of phosphate from water by adsorption using biopolymer chitosan beads. Water Air Soil Pollut..

[17] Wujcicki Ł (2023). Cerium(IV) chitosan-based hydrogel composite for efficient adsorptive removal of phosphates (V) from aqueous solutions scanning electron microscopy with energy dispersive xray spectroscopy. Sci. Rep..

[18] Isiuku BO, Enyoh CE, Duru CE, Ibe FC (2021). Phosphate ions removal from aqueous phase by batch adsorption on activated (activation before carbonization) biochar derived from rubber pod husk. Curr. Res. Green Sustain. Chem..

[19] Brontowiyono W (2022). Phosphate ion removal from synthetic and real wastewater using MnFe_2_O_4_ nanoparticles: A reusable adsorbent. Acta Chim. Slov..

[20] Shahzadi T, Anwaar A, Riaz T, Zaib M (2022). Sulfate and phosphate ions removal using novel nano-adsorbents: Modeling and optimization, kinetics, isotherm and thermodynamic studies. Int. J. Phytoremed..

[21] Paul P, Parbat S, Aditya G (2022). Phosphate ion removal from aqueous solution using snail shell dust: Biosorption potential of waste shells of edible snails. RSC Adv..

[22] Nguyen TT (2022). Effective removal of phosphate from waste water based on silica nanoparticles. J. Chem..

[23] Aboud NAA, Jasim BE, Rheima AM (2021). Adsorption study of phosphate ions pollution in aqueous solutions using microwave synthesized magnesium oxide nanoparticles. Digest J. Nanomater. Biostruct..

[24] Pyrz WD, Buttrey DJ (2008). Particle size determination using TEM: A discussion of image acquisition and analysis for the novice microscopist. Langmuir.

[25] Mohamed SMI, Yılmaz M, Güner EK, El Nemr A (2024). Synthesis and characterization of iron oxide-commercial activated carbon nanocomposite for removal of hexavalent chromium (Cr6+) ions and Mordant Violet 40 (MV40) dye. Sci. Rep..

[26] Khoshnevisan, K. & Barkhi, M. Information about zeta potential. *Encycl. Membr.* 2063–2064 (2015). 10.13140/RG.2.1.4554.3844.

[27] Ferraris S, Cazzola M, Peretti V, Stella B, Spriano S (2018). Zeta potential measurements on solid surfaces for in vitro biomaterials testing: Surface charge, reactivity upon contact with fluids and protein absorption. Front. Bioeng. Biotechnol..

[28] An S, Norlin B, Hummelgard M, Thungström G (2019). Comparison of elemental analysis techniques for fly ash from municipal solid waste incineration using x-rays and electron beams. IOP Conf. Ser. Earth Environ. Sci..

[29] Gong P, Xu M, Ma ZG, Ni XY (2023). Study on pipe-line flow characteristics of multi-source coal-based solid waste filling materials. Therm. Sci..

[30] EPA. Method 365.1, Revision 2.0: Determination of phosphorus by semi-automated colorimetry. *Environ. Monit. Syst. Lab.* 1–15 (1993).

[31] Khandaker S, Toyohara Y, Kamida S, Kuba T (2018). Adsorptive removal of cesium from aqueous solution using oxidized bamboo charcoal. Water Resour. Ind..

[32] Adeogun AI (2018). Biowaste-derived hydroxyapatite for effective removal of reactive yellow 4 dye: Equilibrium, kinetic, and thermodynamic studies. ACS Omega.

[33] Ugoni A, Walker BF (1995). The Chi square test: An introduction. COMSIG Rev..

[34] Molenaar JMM, Katgerman L, Kool WH, Smeulders RJ (1986). On the formation of the stircast structure. J. Mater. Sci..

[35] Schulze TP, Davis SH (1995). Shear stabilization of morphological instability during directional solidification. J. Cryst. Growth.

[36] Liu C, Shih K, Gao Y, Li F, Wei L (2012). Dechlorinating transformation of propachlor through nucleophilic substitution by dithionite on the surface of alumina. J. Soils Sediments.

[37] Habte AT, Ayele DW, Hu M (2019). Synthesis and characterization of reduced graphene oxide (rGO) started from graphene oxide (GO) using the tour method with different parameters. Adv. Mater. Sci. Eng..

[38] Rashidi Nodeh H, Sereshti H, Zamiri Afsharian E, Nouri N (2017). Enhanced removal of phosphate and nitrate ions from aqueous media using nanosized lanthanum hydrous doped on magnetic graphene nanocomposite. J. Environ. Manag..

[39] Ali DA, Sadek MA, Al-Mansi NM (2021). Isotherm and kinetics study for the adsorption of nitrate and phosphate ions from aqueous solutions using fume dust from electric arc furnace. ARPN J. Eng. Appl. Sci..

[40] Ayawei N, Ebelegi AN, Wankasi D (2017). Modelling and interpretation of adsorption isotherms. J. Chem..

[41] Desta MB (2013). Batch sorption experiments: Langmuir and freundlich isotherm studies for the adsorption of textile metal ions onto teff straw (eragrostis tef) agricultural waste. J. Thermodyn..

[42] Kalam S, Abu-Khamsin SA, Kamal MS, Patil S (2021). Surfactant adsorption isotherms: A review. ACS Omega.

[43] Rahman EMA, El-Subruiti GM, Kamel AH, Diab HM, Hassan SSM (2022). Copper and lead ions removal from aqueous solutions case study: Fly ash carbon as low-cost effective sorbent. Egypt. J. Chem..

